# A rare fatal cerebral fat emboli syndrome with large vessel occlusion post femur fracture plating in an older female: A case report

**DOI:** 10.1016/j.ijscr.2024.109828

**Published:** 2024-05-31

**Authors:** Radin H. Kamal, Faldha R. Ramadhan, Marjono Dwi Wibowo, Bimo Sasono

**Affiliations:** a Airlangga University Medical Faculty/Dr. Soetomo General Academic Hospital, Surabaya, East Java, Indonesia; b Dr. Moh. Soewandhie Hospital, Surabaya, East Java, Indonesia

**Keywords:** Rare fatal complication, Cerebral fat emboli syndrome, Large vessel occlusion, Intertrochanter fracture plating, Case report

## Abstract

**Introduction and importance:**

Cerebral fat embolism syndrome is a rare complication of long bone fractures, often overlooked and found in late stages. We present patient with a late recognition of Cerebral Fat Embolism with Large vessel occlusion post femoral internal fixation.

**Case presentation:**

An elderly female suffered right intertrochanteric fracture after falling down. Open reduction internal fixation with Interlocking plate was performed at the fifth day. Upon returning to the ward, the patient did not regain full consciousness and apparent right hemiparesis were observed. A head Computed Tomography was performed and found left hemisphere ischemia consistent with middle cerebral artery occlusion. The patient condition worsened and died 3 days postoperatively.

**Clinical discussion:**

Cases of fat embolism that occur purely isolated in the brain are rare cases that occur after internal fixation of the femur, so they are often not noticed by clinicians. Several factors can increase the risk of the event, delay in fixation and diabetes mellitus which was found in our patient could increase the risk of fat emboli syndrome. Apart from that, osteoporosis also increases the risk of fat embolism syndrome that was found in our subject.

**Conclusions:**

Cerebral Large Vessel Occlusion Fat Embolism Syndrome is rare case occur following internal fixation. There is a need for early recognition to be carried out to treat early or prevent the occurrence of fat embolism.

## Highlights

• Fat embolism in femur fracture is quite rare, isolated fat embolism occurring in the brain without respiratory symptoms is even rarer, for this reason we present this case to increase awareness of cases of femur fracture in patients at risk
• Early immobilization is one of the preventive managements that could be taken to prevent fat embolism in femur fracture patients, delaying action can increase the risk of fat embolism incident
• Postoperative observation is highly recommended in emergency and elective cases, because fat embolism can happen to anyone and early treatment can improve the patient's prognosis

## 1 Introduction and importance

Fat embolism is a condition that is described as the dissemination of fat emboli in the blood vessel circulation. The incidence of Fat Embolism Syndrome (FES) in trauma cases, especially femur fractures, is quite rare, but has the mortality rate ranged between 7% and 36 % [1,2]. The diagnosis of FES is by clinical and scoring, although in some cases scoring is not that sensitive [3]. Cerebral FES (CFES) has a much lower incidence where there are no systemic but neurological symptoms [[3], [4], [5]].

In CFES in which a large fat emboli that occlude major cerebral arteries is termed Cerebral Large Vessel Occlusion Fat Embolism Syndrome (CLVOFES) [3]. CLVO due to fat embolism is far less common than CFES, leading to a lack of general awareness. In this case report, we report a case of cerebral fat embolism with large vessel occlusion in a post internal fixation of a femoral fracture patient, work has been reported in line with the Surgical Case Report (SCARE) guidelines criteria [6].

## 2 Case presentation

**Fig. 1:**
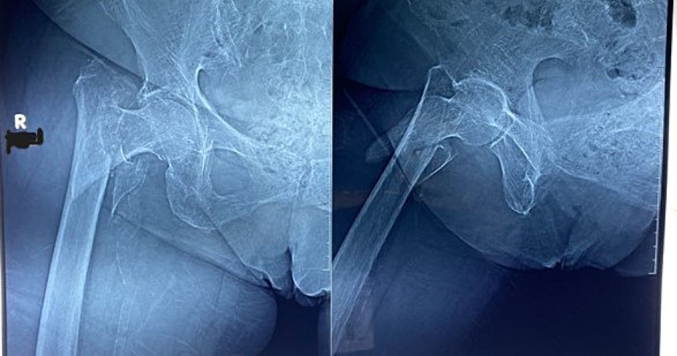
Plain Xray of right femur Boyd Griffin type II Classification intertrochanteric fracture.

**Fig. 2:**
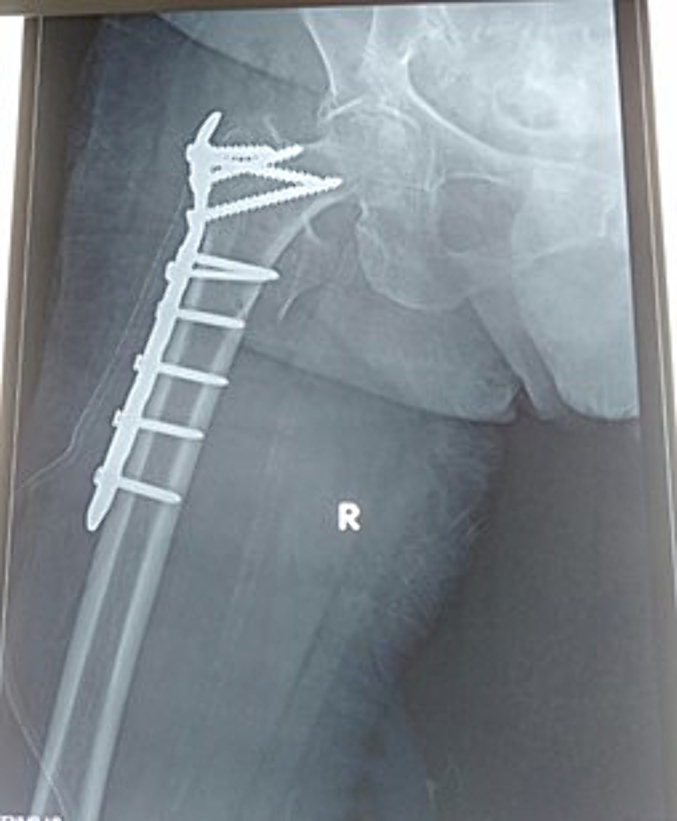
Plain Xray of post intertrochanteric plating with interlocking plate.

We presented elderly 69-year-old diabetic and hypertensive female with a right limb deformity and false movement after stumbling in front of the house. Patient was unoccupied with GIR score 3. General status was well, with BMI 27,05, no abnormalities on head, neck, chest, abdomen, pelvis nor superior extremities were observed. On the right hip showed a 3 × 3 cm bruises, externally rotated, slight edema of the right outer hip. The hip joint could not be moved due to pain on movement, without evidence of neurological deficits, from femur x-ray showed discontinuity at femur trochanter and assessed with right femur Boyd Griffin type II Classification intertrochanteric fracture (Fig. 1). The patient had prior history of diabetes and hypertension, without hyperlipidemia with routine medication of Metformin and Candesartan. Chest X-ray and echocardiography were within normal limit. The fracture was stabilized using skin traction and Open reduction internal fixation with Interlocking plate using 3 Cancellous Screws and 5 Cortical Screw was performed on the fifth day (Fig. 2). The operation using general anesthesia with the duration of the operation was 60 min and significant blood loss was not observed. The patient was stable during and post-operative observation.

**Fig. 3:**
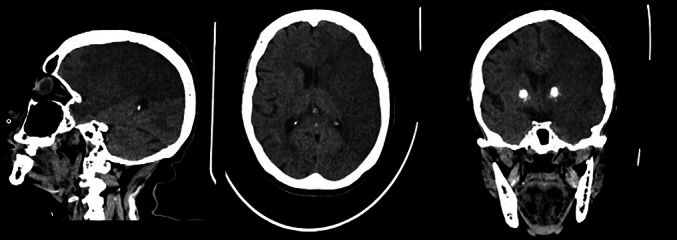
Head CT scan acute-subacute cerebral infarction in the cortical-subcortical area, prominent calcification of the right and left basal ganglia and calcification of the right and left vertebral arteries consistent with cerebral large vessel occlusion of the left middle cerebral artery.

Upon returning to the ward, the patient did not regain full consciousness and apparent right hemiparesis were observed. General status was within normal limit, and petechial rash was not found neither classical sign of fat emboli syndrome was not evident. Observations in the ICU on the first day showed that the patient was in stable vital sign with a GCS of E3V2M5, high blood pressure (150/100 mm Hg) blood sugar was high and without evidence of other abnormalities on physical examination. Imaging and laboratory examination were within normal limit, consistent with preoperative results, and showed neither anemia, thrombocytopenia, hyperbilirubinemia, hypoxia, hypercarbia nor acidemia (Table 1). Lateralization was found on the patient's right side. Head CT without contrast found an acute-subacute cerebral infarction in the cortical-subcortical area (Fig. 3), prominent calcification of the right and left basal ganglia and calcification of the right and left vertebral arteries consistent with cerebral large vessel occlusion of the left middle cerebral artery. The patient was then treated in the intensive care room 6 hours after diagnostics. The patient condition worsened even after treatment with interdisciplinary team, the patient treated by palliative care and transferred back to low care room. The patient died 2 days after discharged from intensive care room, the family was informed and accepted. The timeline presented at Table 2.

**Table 1:** Laboratory examination during patient admission (26/08/2023).

Examination	Value	Unit
Hemoglobin	11.2	g/dL
Erytrocyte	3.69	10^6/uL
Hematocrite	34.3	%
Leukocyte	11.24	10^3/uL
Eosinophil	0.0	%
Basophil	0.4	%
Neutrophil	81.8	%
Lymphocyte	8.6	%
Monocyte	9.2	%
PT	9.8	Seconds
APTT	21.4	Seconds
INR	0.88	
SGOT	13	U/L
SGPT	18	U/L
Natrium	138	Mmol/L
Kalium	4.2	Mmol/L
Creatinine	1.2	Mg/dL
Glucose	352	Mg/dL

**Table 2:** Patient timeline.

Day (date)	0 (26/08/2023)	5 (31/08/2023)	6 (01/09/2023)	7 (02/09/2023)	9 (04/09/2023)
Event	Patient after stumbled in front of her house came to emergency ward and skin tracted, and scheduled for internal fixation femur O: GCS E4V5M6 Blood pressure (BP): 153/74 Heart rate (HR): 98 x/m Respiratory rate (RR): 22 x/m Temperature: 36,5 SpO2: 97 % free air	Patient fracture was done internal fixation using general anesthesia, no complication during the procedure. Patient still unconscious after general anesthesia O: BP: 121/88 Temp: 36.4 HR: 87 RR: 18 SpO2 98 % with O2 3 l/min	Patient did not regain her consciousness after transferred to low care ward, patient than consulted to intensive care and neurology, patient transferred to Intensive Care Unit and done head CT scan O: GCS E3V2M5 BP: 117/90 Temp: 36.5 HR: 83 RR: 22 SpO2 97 % with O2 3 l/min Blood glucose: 309 mg/dL	Patient still unconscious and advised to do tracheal intubation to secure patient airway but rejected by the family considering patient age and comorbidities. Patient planned transfer back to low care ward and planned for palliative care O: GCS E3V1M4 BP: 117/90 Temp: 36.5 HR: 83 RR: 24 SpO2 100 % with O2 3 l/min	Patient treated at low care ward, the condition was deteriorated and patient declared dead at 13.45

## 3 Clinical discussion

The incidence of fat embolism in femoral fracture cases is classified as a rare event from 1.7 % to 7.9 % [7] and has a high mortality rate of 17.6 %, so it requires special attention when finding signs and symptoms of fat embolism manifestations [1]. More frequent in men than women [8]. Symptoms of fat embolism vary from dermatological, respiratory to neurological manifestations [9]. Cerebrovascular accident is one of the major complications of fat embolism which can threaten the patient's life, which occurred in our case. The mortality rate from embolic stroke is quite large, reaching 23 %, in which in our case the patient had died [10].

The main hypothesis of fat embolism in fracture is due to three possible mechanisms; mechanical, biochemical, and combined. External manipulation which might contribute to fat dislodge from the intramedullary to the intravascular [9]. Delayed stabilization for femur fractures increases the incidence of acute respiratory distress syndrome (ARDS) and fat embolism [11]. Metabolic stress triggers catecholamines release, activation of adenylyl cyclase pathway, lipase activation, hydrolyzing triglycerides into free fatty acids and glycerol which then enter the systemic circulation, and also microvascular obstruction free fatty acid mediated endothelial injury and excess thrombin, elevated tissue factor fibrin generation and consumption of coagulation product may lead to ARDS, encephalopathy, and focal neurological deficit, cutaneous petechiae, DIC, thrombocytopenia, and anemia [2,12,13]. Diabetes mellitus is a risk factor for fat embolism [14] and a hazard ratio of ischemic stroke of 2.27 times even with minimal impact on soft tissue [2,15]. Hypertension also increases the risk of fat embolism formation [16]. Fractures with minimal impact have a low risk of fat embolism, but not in cases of pathological fractures, especially due to osteoporosis [17,18]. In the case of osteoporosis, bone tissue is substituted with fat tissue resulting in more fat being dislocated intravascularly and increasing the risk of fat embolism [1,19]. The diagnosis of FES is based on clinical and laboratory findings. Three different criteria proposed by Gurd, Schonfeld, and Lindeque [20] did not meet the requirements of FES because only cerebral symptoms appeared in the patient. Our report can strengthen the case that the occurrence of cerebral fat embolism may not always be accompanied and is a lot harder to diagnose compared to classic FES. [4,21]. Because of the high load of urgent surgery queue, internal fixation was performed on the fifth day, but the patient's fracture was stabilized with skin traction from the first day.

CFES is an incomplete type of FES that described by the incidence of purely cerebral symptoms that occur 12–72 h after initial trigger, which in this case is intertrochanteric fracture plating [4,22]. Large cerebral infarcts involving the major arteries of the brain almost likely are caused extra-cranial emboli. Clinical manifestations are also more severe compared to thrombosis [23]. Fat emboli may gain access to systemic circulation that can affect heart, brain, skin, and retina with possible explanation of: (1) Pulmonary A-V malformation, (2) Passing of deformed fat globule through pulmonary capillaries, (3) bypassing pulmonary circulation via patent foramen ovale, (4) re-opening of close foramen ovale due to acute rise in pulmonary pressure [24]. A previous case series on the incidence of cerebrovascular abnormalities after major orthopedic surgery found occlusion of the middle cerebral artery due to fat occlusion accompanied by fibrin nets indicating the presence of fat embolism [3]. In our case, infarction of the temporoparietal cortical and subcortical areas, consistent with large vessel occlusion of the left middle cerebral artery. There were no other complications from FES such as respiratory or dermatologic which commonly appear in FES cases so that clinically it is difficult to recognize, so observation should be carried out in cases of patients who are at risk of having FES. It is important to evaluate the risk factors before surgery and observe the patient's neurological status and consciousness post-operatively because there is no obvious sign presented by the patient [3].

Recent papers showed early diagnosis was possible, and may increase the chance of survival. Large vessel occlusion of the major vessels' evacuation and reperfusion was able to be performed using endovascular clot retrieval, even with severe morbidity and sequalae [3]. CFES should be suspected even 72 h after treatment, especially in patients who develop decrease of consciousness [21]. Late recognition of the neurological disorders delayed proper treatment for several hours. The decline in neurological status was not previously detected because the patient was in a sedated condition post-operatively. Suspicions arose when there was no improvement in the patient's consciousness which is similar to published cases. Observation and the familiarity of clinical symptoms of CFES should alert all healthcare personnel as sometimes recovering from an anesthesia and CFES symptoms might be hard to differentiate.

Although no commonly accepted guidelines for CFES, especially CLVOFES, general precaution and management of FES is still used. Preoperative prevention in early internal fixation can reduce the risk of FES (Scarpino et al., 2019). Intraoperative prevention by managing the increase in intramedullary pressure due to mechanical compression caused by internal fixation or by slow insertion to the bone and distal venting, beside that change reamer design include narrowing the reamers could reduce pressure may increase the risk of emboli development [25]. Corticosteroids and heparin were considered to have a role in preventing the formation of fat emboli in patients undergoing major orthopedic surgery. In daily elective cases, heparin or corticosteroids can be given before surgery in patients with a risk of fat embolism, but in trauma cases, where bleeding has already occurred, giving heparin can make the patient's bleeding worse [4,13]. Apart from that, post-operative observation, especially in groups at risk, is also needed to avoid treatment delay in cases of CFES.

## 4 Conclusion

Cerebral Large Vessel Occlusion Fat Embolism Syndrome is a rare complication of post intertrochanteric fracture plating. Our case showed a delayed recognition which lead to a fatal outcome. In retrospect, with a high clinical suspicion of CLOVES might be the main prevention of further morbidity and might have led to a better outcome due to early initiation of treatment. Good rapport and preoperative education to patients and family members about the possibility of CLOVES or other spectrum of FES should be done. Although supportive care is still the gold standard of treatment, early operation, careful technique and excellent perioperative care can lead to decrease risk of CLOVES incidence. Due to its rarity, all clinician, especially surgeon and orthopedic surgeon must be familiar and well versed in recognition and promotion of good clinical practices of pre, intra and postoperative monitoring for early signs of FES and especially CLOVES.

## Consent

Written consent was obtained from the patient's family for publication and any accompanying images. A copy of the written consent is available for review by the Editor in Chief of this journal by request.

## Ethical approval

Ethics approval was not required for this study. Consent had been obtained from subject's family.

## Funding

This research not received any funding from third party.

## CRediT authorship contribution statement

Radin H Kamal: Study design, Data Collection, Writing.

Faldha R Ramadhan: Writing, Analytic, Data Collecting.

Bimo Sasono: Study design, Supervision, Conceptualization, Head Project.

Marjono D Wibowo: Study design, Supervision, Conceptualization.

## Guarantor

Bimo Sasono.

## Research registration number

1. Name of the registry:

2. Unique identifying number or registration ID:

3. Hyperlink to your specific registration (must be publicly accessible and will be checked):

## Conflict of interest statement

The authors declare that they have no known competing financial interests or personal relationships that could have appeared to influence the work reported in this article.
